# Tip perfusion index for monitoring perioperative stress responses in patients undergoing laparoscopic cholecystectomy: a prospective cohort study

**DOI:** 10.3389/fmed.2026.1921275

**Published:** 2026-10-02

**Authors:** Xiaolin Wang, Jingjing Zhang, Sufang Jiang, Jiaojiao Yang, Ya Liu

**Affiliations:** 1 Department of Anesthesiology, Shijiazhuang People’s Hospital, Shijiazhuang, China; 2 Department of Anesthesiology, The Second Hospital of Hebei Medical University, Shijiazhuang, China

**Keywords:** hypertension, intraoperative care, laparoscopic cholecystectomy, microcirculation, sympathetic activation, type 2 diabetes mellitus

## Abstract

**Objective:**

This study aimed to characterize peri-stimulus changes in the Tip Perfusion Index (TPI) and conventional hemodynamic parameters during elective laparoscopic cholecystectomy and to compare their response dynamics among patients without major comorbidities, patients with hypertension, and patients with type 2 diabetes mellitus.

**Methods:**

Ninety patients undergoing elective laparoscopic cholecystectomy were prospectively enrolled and classified as Control, Hypertension, or Diabetes (*n* = 30 each). TPI, BP, and HR were recorded at six perioperative time points (T0–T5) under standardized general anesthesia and CO2 pneumoperitoneum. In a randomly selected subcohort (*n* = 5 per group), plasma E, NE, and COR were measured from T0 to T4. Data were analyzed using Chi-square tests, one-way ANOVA, mixed-design repeated-measures ANOVA, and repeated-measures correlation.

**Results:**

Demographic and baseline parameters were comparable among the three groups (*P* > 0.05). Following endotracheal intubation and pneumoperitoneum, TPI decreased from the corresponding pre-stimulus values, whereas blood pressure and heart rate increased. Mixed-design repeated-measures ANOVA showed significant Group×Time interactions for DBP (*P* = 0.030) and HR (*P* = 0.002), but not for TPI (*P* = 0.069) or SBP (*P* = 0.619). Repeated-measures correlation showed inverse associations between TPI and hemodynamic parameters and stress hormones, except for cortisol in the Diabetes group, where the association did not reach statistical significance (*P* = 0.057). In the separate threshold-based analysis, all participants met the predefined TPI-positive response criterion during both stimuli. During pneumoperitoneum, TPI response time differed significantly among the three groups (*P* < 0.001), with the longest mean response time observed in the Diabetes group, whereas no significant between-group differences in response time were observed for SBP, DBP, or HR (all *P* > 0.05).

**Conclusion:**

TPI showed consistent peri-stimulus changes during endotracheal intubation and pneumoperitoneum and may provide complementary information on peripheral vascular responses during surgical stress. The prolonged threshold-based TPI response time observed in patients with diabetes during pneumoperitoneum suggests a potential difference in response timing that warrants further investigation.

## 1 Introduction

Surgical procedures trigger neuroendocrine and physiological stress responses, largely mediated by activation of the sympathetic-adrenal-medullar*y* axis (1, 2). In clinical anesthesia, balancing nociceptive stimulation and antinociceptive management is important for maintaining hemodynamic stability and perioperative safety (3). Blood pressure (BP) and heart rate (HR) are commonly used to monitor physiological responses to surgical stimulation (4, 5). However, these systemic hemodynamic variables are influenced by anesthetic depth, vasoactive medications, baseline cardiovascular status, and compensatory mechanisms, which may limit their ability to reflect rapid changes in peripheral vascular tone (6, 7). Therefore, additional non-invasive monitoring variables that provide real-time information on peripheral perfusion may be clinically useful.

The tip perfusion index (TPI), extracted non-invasively via photoelectric plethysmography from the digital pulse oximetry waveform, has recently emerged as a promising surrogate modality for assessing peripheral vascular tone (8, 9). TPI is mathematically defined as the ratio of the pulsatile component (arterial blood flow) to the non-pulsatile component (static tissues and venous blood) of light absorption, serving as a direct reflection of regional microcirculatory perfusion (10). Because the cutaneous microvasculature of the fingers is richly influenced by sympathetic vasomotor activity, nociceptive stimulation may induce peripheral vasoconstriction and a corresponding decrease in TPI (11). Compared with systemic BP or HR, TPI may provide more direct information on changes in peripheral vascular tone and perfusion during acute physiological stress (12). Nevertheless, its comparative warning efficacy and sensitivity profiles require extensive clinical clarification across varied patient phenotypes.

Laparoscopic cholecystectomy provides a relatively standardized clinical setting for evaluating perioperative stress responses because it includes two distinct noxious stimuli: endotracheal intubation and CO₂ pneumoperitoneum (13, 14). Hypertension and type 2 diabetes mellitus are common comorbidities among surgical patients and may influence vascular and autonomic responses to perioperative stimulation (15). Hypertension is associated with vascular remodeling and endothelial dysfunction (16), whereas type 2 diabetes may be accompanied by microvascular dysfunction, impaired nitric oxide bioavailability, and autonomic abnormalities (17, 18). These comorbidity-related alterations could modify the magnitude or timing of peripheral vascular responses during surgical stress and therefore warrant consideration when interpreting TPI changes.

To date, few clinical investigations have systematically mapped the parallel responsiveness and temporal warning thresholds of TPI against both macrohemodynamic variables and neuroendocrine stress markers—specifically plasma epinephrine (E), norepinephrine (NE), and cortisol (COR)—within hypertensive and diabetic subpopulations. To bridge this critical knowledge gap, this prospective cohort study was rigorously designed to compare the dynamic sensitivity, precise warning response times, and stress biomarker correlations of TPI among healthy controls, hypertensive patients, and diabetic individuals undergoing laparoscopic cholecystectomy. The study was designed to determine whether TPI exhibits distinct peri-stimulus response patterns compared with conventional hemodynamic parameters and whether these response dynamics differ among healthy controls, patients with hypertension, and patients with type 2 diabetes mellitus.

## 2 Methods

### 2.1 Ethical approval and patient selection

This prospective observational study was conducted at the Second Hospital of Hebei Medical University between June 2020 and February 2021. The study protocol was approved by the Medical Ethics Committee of the Second Hospital of Hebei Medical University (approval no. 2020-R384), and written informed consent was obtained from all participants before enrollment.

A total of 97 patients scheduled for elective laparoscopic cholecystectomy were initially screened for eligibility. Following the rigorous screening and enrollment flow, 90 patients were ultimately selected and tightly allocated into three parallel cohorts (*n* = 30 per group): the healthy Control Group (Group N), the Hypertension Group (Group H), and the Type 2 Diabetes Mellitus Group (Group D). Figure 1 visually schematizes the detailed recruitment dynamics and specific exclusion reasons.

**Figure 1:**
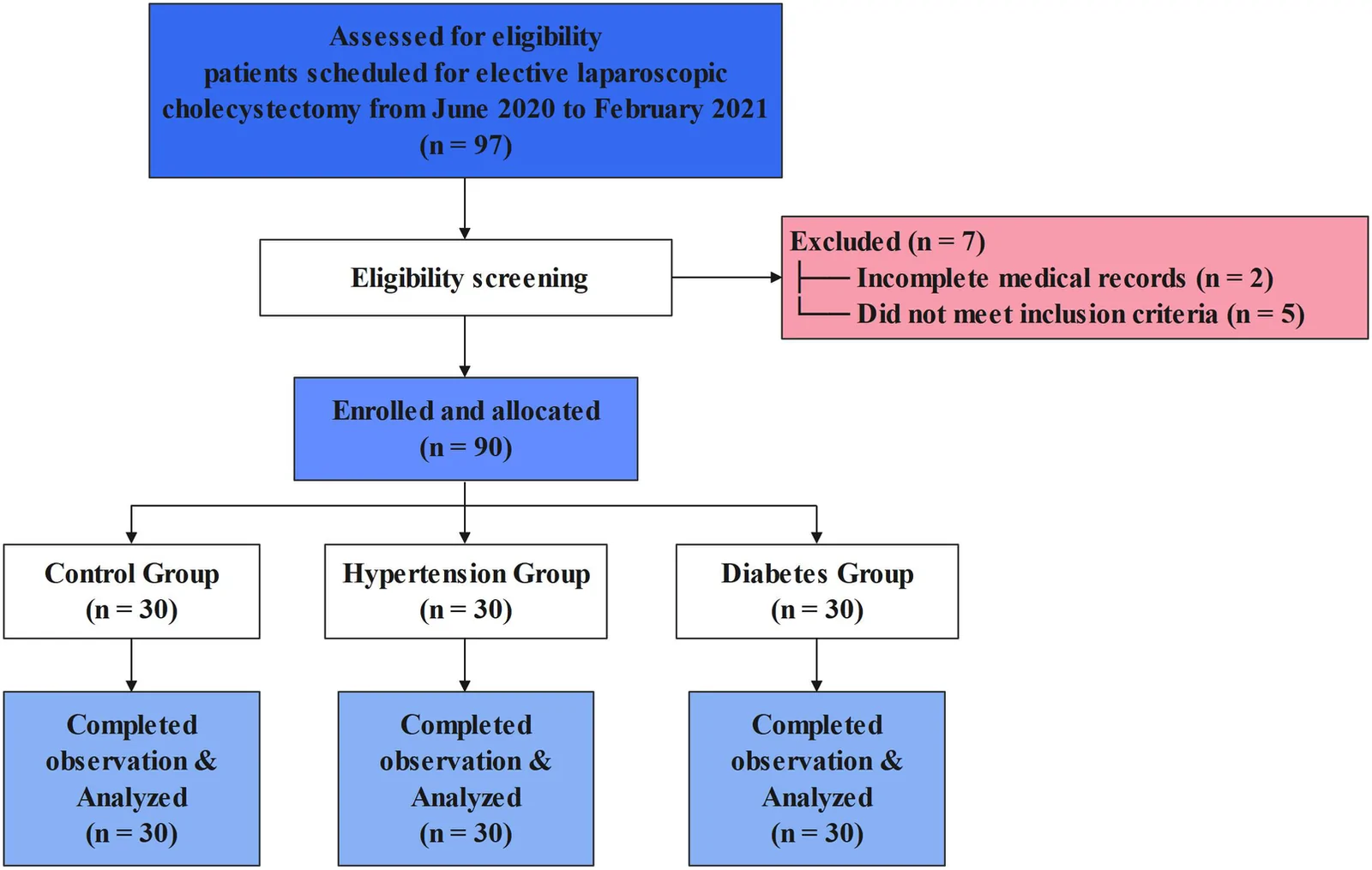
Flowchart of the study.

### 2.2 Inclusion and exclusion criteria

#### 2.2.1 Inclusion criteria

- Scheduled for elective laparoscopic cholecystectomy under standardized general anesthesia;
- Aged between 18 and 65 years, with a body mass index (BMI) restricted within 18–28 kg/m^2^;
- Possessing an American Society of Anesthesiologists (ASA) physical status of I or II;
- For Group H, a confirmed medical history of essential hypertension under stable, regular antihypertensive medication tracking, with preoperative blood pressure optimized within clinically acceptable surgical target ranges;
- For Group D, a clinical diagnosis of type 2 diabetes mellitus with documented preoperative glycated hemoglobin (HbA1c) levels restricted between 7.0% and 8.5%.

#### 2.2.2 Exclusion criteria

- A documented history of trauma, vascular anomalies, or prior surgical interventions in the upper limbs;
- Pre-existing primary autonomic nerve disorders or severe neurological entities;
- Extensive abdominal adhesions anticipated to severely prolong the procedure or necessitate open conversion;
- Fluctuations in perioperative core body temperature fell below 36.0°C or exceeded 37.5°C;
- Intraoperative administration of any vasoactive or chronotropic medications that heavily affect blood pressure or heart rate, such as atropine or ephedrine, during the anesthesia induction or maintenance phase, outside the scope of the standardized protocol.

### 2.3 Anesthesia protocol and monitoring

Upon arrival in the operating room, standard monitoring was established, including non-invasive blood pressure (NIBP), electrocardiography (ECG), pulse oxygen saturation (SpO_2_), and body temperature monitored via a nasopharyngeal probe. TPI and HR were monitored continuously. NIBP was obtained intermittently using an automated oscillometric cuff. During the periods surrounding anesthesia induction and endotracheal intubation, the NIBP measurement interval was shortened to approximately 1 min and was returned to approximately 3 min after the immediate post-intubation period. Before pneumoperitoneum, the measurement interval was again shortened to approximately 1 min and was returned to approximately 3 min after the immediate post-pneumoperitoneum observation period. Additional NIBP measurements could be obtained when clinically indicated. TPI was monitored using a Masimo SET® pulse oximeter (Masimo Corp., Irvine, CA, USA) securely attached to the left index finger. Before formal data collection, a brief deep-breath maneuver was performed to confirm an observable change in the peripheral perfusion signal. Absence of any PI change was prespecified as an exclusion criterion; however, no screened participant was excluded on this basis (19). Bispectral index (BIS) sensors were applied to the forehead after skin preparation.

An intravenous line was established, and 500 mL of lactated Ringer's solution was infused before anesthesia induction. Anesthesia was induced with midazolam (0.02 mg/kg), etomidate (0.2 mg/kg), sufentanil (0.5 μg/kg), and cisatracurium (0.15 mg/kg). Following tracheal intubation, mechanical ventilation was initiated with a tidal volume of 6–10 mL/kg and a respiratory rate of 12 breaths/minute. Ventilatory parameters were adjusted after pneumoperitoneum establishment to maintain end-tidal carbon dioxide (PETCO_2_) between 35 and 45 mmHg. Anesthesia was maintained with continuous infusions of remifentanil (0.1–0.3 μg/kg/min) and propofol (4–12 mg/kg/h), with infusion rates titrated within these predefined ranges to maintain BIS values between 40 and 60. The same standardized anesthesia protocol was applied to all three study groups. Cumulative propofol doses (mg) and remifentanil doses (μg) recorded at predefined perioperative time points were subsequently compared among the three groups to assess potential between-group differences in anesthetic drug exposure. CO₂ pneumoperitoneum was established and maintained at an intra-abdominal pressure of 12 mmHg throughout the procedure.

### 2.4 Data collection

Baseline demographic and clinical characteristics, including sex, age, height, weight, antihypertensive medication class, available information on antidiabetic treatment, and documented diabetes-related complications, were extracted from the preoperative medical records. Macro-hemodynamic parameters, including systolic blood pressure (SBP), diastolic blood pressure (DBP), and HR, together with TPI, were recorded at six predefined perioperative time points: baseline upon operating room entry (T0), 1 min before endotracheal intubation after anesthesia induction (T1), immediately after endotracheal intubation (T2), 1 min before pneumoperitoneum (T3), 1 min after establishment of CO₂ pneumoperitoneum (T4), and 5 min after pneumoperitoneum (T5). In addition, cumulative propofol doses (mg) recorded at T3, T4, and T5 and cumulative remifentanil doses (μg) recorded at T2, T3, T4, and T5 were collected for between-group comparison of perioperative anesthetic drug exposure.

To quantify the physiological response to each noxious stimulus, a positive stress response was defined relative to the corresponding pre-stimulus reference value, using T1 for endotracheal intubation and T3 for pneumoperitoneum. A positive response was considered present when HR increased by ≥10 beats/minute, SBP or DBP increased by ≥15 mmHg, or TPI decreased by ≥10% from the corresponding pre-stimulus value. These parameter-specific thresholds were used as operational criteria for within-parameter response detection and were not assumed to represent physiologically equivalent or validated sensitivity thresholds across TPI, blood pressure, and heart rate (20–22). Response time was defined as the interval from the onset of the corresponding surgical stimulus (endotracheal intubation or pneumoperitoneum) to the first attainment of the predefined response threshold. For continuously monitored TPI and HR, the response time was determined from the continuous monitoring record. For SBP and DBP, the response time corresponded to the first intermittent NIBP measurement at which the predefined threshold was reached. Consequently, the temporal resolution of SBP and DBP response-time measurements was limited by the NIBP measurement interval.

For the neuroendocrine stress profile evaluation, five patients were randomly selected from each study group, resulting in a total subcohort of 15 patients. Each selected patient underwent longitudinal blood sampling at five consecutive perioperative time points from T0 through T4, yielding five repeated measurements per patient. At each time point, a 3 mL sample of peripheral venous blood was collected through the established intravenous line into an ethylenediaminetetraacetic acid (EDTA) anticoagulant tube. The samples were gently mixed, maintained at 4°C for 10–20 min, and subsequently centrifuged at 2,000 r/min for 20 min. The isolated plasma supernatant was aliquoted and stored at −80°C until batch analysis. Plasma concentrations of epinephrine (E), norepinephrine (NE), and cortisol (COR) were measured using commercially available enzyme-linked immunosorbent assay (ELISA) kits (Jiangsu Jingmei Biotechnology Co., Ltd., Jiangsu, China) in accordance with the manufacturer's instructions. The catalog numbers and lot-specific intra-assay and inter-assay coefficients of variation could not be reliably retrieved from the archived study records and are therefore not reported.

### 2.5 Statistical analysis

All statistical analyses were performed using IBM SPSS Statistics (version 25.0). The normality of continuous variables was assessed using the Shapiro–Wilk test. Normally distributed continuous variables are presented as mean ± standard deviation $\left(\bar{x}\pm s\right)$, whereas non-normally distributed variables are presented as median and interquartile range.

Baseline demographic and clinical characteristics were compared among the three groups using one-way analysis of variance (ANOVA) for normally distributed continuous variables and the Chi-square test or Fisher's exact test for categorical variables, as appropriate. Cumulative propofol doses (mg) at T3, T4, and T5 were compared among the three groups using one-way ANOVA and are presented as mean ± standard deviation. Cumulative remifentanil doses (μg) at T2, T3, T4, and T5 showed non-normal distributions and were therefore compared using the Kruskal–Wallis test and are presented as median and interquartile range. Longitudinal perioperative changes in TPI, SBP, DBP, and HR were analyzed using a mixed-design repeated-measures ANOVA, with Group (Control, Hypertension, and Diabetes) specified as the between-subject factor and Time (T0–T5) as the within-subject factor. The main effects of Group and Time, as well as the Group-by-Time interaction, were evaluated. Greenhouse–Geisser correction was applied to the within-subject Time effect and the Group-by-Time interaction where appropriate. Planned within-group comparisons included T1 vs. T0, T2 vs. T1, T3 vs. T0, T4 vs. T3, and T5 vs. T3, with Bonferroni adjustment applied for multiple comparisons. For supplementary between-group comparisons at individual perioperative time points, one-way ANOVA was also performed, as presented in Table 2. These cross-sectional comparisons were considered secondary to the longitudinal repeated-measures analysis.

Positive stress response rates were compared using the Chi-square test or Fisher's exact test, as appropriate. Among participants who reached the predefined response threshold, response times were compared among the three groups using one-way ANOVA. Response-time analyses were restricted to participants who reached the predefined response threshold for the corresponding parameter and stimulus. Participants who did not reach the threshold were retained in the positive-response rate analysis but were not included in the calculation or between-group comparison of response times. Accordingly, response-time results were interpreted as conditional on having achieved the predefined response criterion rather than as estimates applicable to the entire study group.

Associations between TPI and SBP, DBP, HR, epinephrine (E), norepinephrine (NE), and cortisol (COR) were assessed using repeated-measures correlation (r_rm), with participant treated as the repeated-measures unit to account for within-subject dependence. Hemodynamic analyses included 30 patients per group with six repeated measurements per patient, whereas neuroendocrine analyses included five patients per group with five repeated measurements per patient.

All statistical tests were two-sided, and a *P* value < 0.05 was considered statistically significant.

### 2.6 Sample size calculation

The original sample-size estimation was performed using PASS software (version 15.0) and was based on the expected proportion of participants meeting the predefined TPI-positive response criterion after noxious stimulation. Based on previously reported data, the expected TPI-positive response proportion was 100%, with a target confidence-interval width of 0.176 (22). Under these assumptions, the minimum required sample size was approximately 20 participants per group. To allow for up to 20% potential loss or incomplete data, the planned enrollment target was increased to at least 25 participants per group.

The final analyzed cohort included 30 participants in each group, for a total of 90 participants. No post-enrollment attrition occurred among these 90 participants; therefore, the final sample exceeded the minimum planned enrollment target. The neuroendocrine subcohort was exploratory and was not separately powered for definitive between-group comparisons.

## 3 Results

### 3.1 General information and baseline characteristics of patients

A total of 90 patients were enrolled and successfully completed the study, with 30 participants in each cohort. As shown in Table 1, there were no statistically significant differences in demographic variables, including age, gender distribution, height, weight, and BMI, among the Control, Hypertension, and Diabetes groups (*P* > 0.05).

**Table 1:** Demographic and baseline clinical characteristics of patients among the three groups.

Characteristic	Control Group (*n* = 30)	Hypertension Group (*n* = 30)	Diabetes Group (*n* = 30)	Statistical test values	*P* value
Age ($\bar{x}\pm s$, years)	51.73 ± 8.44	55.57 ± 8.78	52.67 ± 11.08	1.327	0.271
Gender [n(%)]				1.216	0.545
Men	10 (33.3%)	14 (46.67%)	11 (36.67%)		
Women	20 (66.7%)	16 (53.33%)	19 (63.33%)		
Height ($\bar{x}\pm s$, cm)	164.03 ± 5.97	167.43 ± 9.73	165.43 ± 8.46	1.302	0.277
Weight ($\bar{x}\pm s$, kg)	63.88 ± 10.98	69.07 ± 11.39	66.68 ± 13.05	1.440	0.243
BMI ($\bar{x}\pm s$, kg/m^2^)	23.63 ± 3.22	24.56 ± 2.88	24.19 ± 3.39	0.654	0.523
Baseline parameters at T0					
Baseline SBP ($\bar{x}\pm s$, mmHg)	129.30 ± 7.53	132.87 ± 8.23	130.97 ± 12.23	1.047	0.355
Baseline DBP ($\bar{x}\pm s$, mmHg)	79.37 ± 7.24	84.53 ± 8.20	81.60 ± 10.98	2.515	0.087
Baseline HR ($\bar{x}\pm s$, bpm)	80.07 ± 9.88	73.83 ± 9.43	75.73 ± 11.78	2.824	0.065
Baseline TPI $\left(\bar{x}\pm s\right)$	2.49 ± 1.57	2.57 ± 0.80	2.52 ± 1.02	0.039	0.962

Regarding the hemodynamic parameters measured upon arrival in the operating room (T0), the baseline SBP, DBP, and HR were comparable across the three groups, with no statistical variance observed (*P* = 0.355, 0.087, and 0.065, respectively). Furthermore, the baseline TPI at the T0 time point showed no significant differences among the healthy control, hypertensive, and diabetic populations (2.49 ± 1.57, 2.57 ± 0.80, and 2.52 ± 1.02, respectively, *P* = 0.962). Overall, no statistically significant baseline differences were observed among the three groups in the measured demographic and hemodynamic characteristics.

Regarding comorbidity-related clinical characteristics, antihypertensive therapy in Group H consisted of calcium channel blockers (CCBs) in 24 patients, angiotensin-converting enzyme inhibitors (ACEIs) in 4 patients, and angiotensin receptor blockers (ARBs) in 2 patients. In Group D, peripheral neuropathy was documented in 2 patients. Information on antidiabetic treatment was incompletely documented, and diabetes duration was not consistently available in the archived records; therefore, these variables were not included in the formal comparative analyses.

### 3.2 Perioperative anesthetic exposure

As shown in Table 2, cumulative propofol doses (mg) and remifentanil doses (μg) recorded at the predefined perioperative time points were compared among the three groups. No statistically significant between-group differences were detected in cumulative propofol doses at T3, T4, or T5 (all *P* > 0.05). Similarly, cumulative remifentanil doses at T2, T3, T4, and T5 did not differ significantly among the Control, Hypertension, and Diabetes groups (all *P* > 0.05).

**Table 2:** Comparison of cumulative perioperative propofol and remifentanil exposure Among the three study groups.

Anesthetic exposure	Control Group (*n* = 30)	Hypertension Group (*n* = 30)	Diabetes Group (*n* = 30)	Statistical test values	*P* value
Propofol cumulative dose at T3 (mg)	42.97 ± 7.38	45.98 ± 7.64	44.30 ± 8.68	1.091	0.340
Propofol cumulative dose at T4 (mg)	64.21 ± 11.00	68.97 ± 11.41	66.47 ± 13.02	1.212	0.302
Propofol cumulative dose at T5 (mg)	85.49 ± 14.58	91.96 ± 15.19	88.64 ± 17.35	1.264	0.288
Remifentanil cumulative dose at T2 (μg)	100.0 (100.0–100.0)	100.0 (100.0–100.0)	100.0 (100.0–100.0)	1.107	0.575
Remifentanil cumulative dose at T3 (μg)	164.5 (155.0–173.8)	168.5 (160.0–175.4)	166.5 (157.0–180.0)	2.996	0.224
Remifentanil cumulative dose at T4 (μg)	296.1 (279.0–312.8)	303.3 (288.0–315.7)	299.7 (282.6–324.0)	2.996	0.224
Remifentanil cumulative dose at T5 (μg)	347.7 (323.0–371.8)	358.1 (336.0–376.0)	352.9 (328.2–388.0)	2.996	0.224

Data are presented as mean ± standard deviation for propofol and median (interquartile range) for remifentanil. Propofol cumulative doses are reported in mg and remifentanil cumulative doses in μg. At T2, although the median and interquartile range of remifentanil dose were identical across the three groups, individual values were not identical (range: 60–100 μg in the Control group and 80–100 μg in both the Hypertension and Diabetes groups), resulting in a non-zero Kruskal–Wallis statistic.

At T2, the median (interquartile range) of cumulative remifentanil dose was 100.0 (100.0–100.0) μg in all three groups. However, the individual-level data were not identical: the observed ranges were 60–100 μg in the Control group and 80–100 μg in both the Hypertension and Diabetes groups. Therefore, the non-zero Kruskal–Wallis statistic (H = 1.107, *P* = 0.575) reflects differences in the underlying rank distributions that are not apparent from the identical median and interquartile-range summaries.

Overall, no statistically detectable between-group imbalance in the recorded anesthetic drug exposure was observed at the assessed perioperative time points.

### 3.3 Dynamic profiles of hemodynamics and TPI

The dynamic trajectories of TPI and hemodynamic parameters throughout the perioperative period are illustrated in Figure 2, with descriptive data presented in Table 3 and the repeated-measures analysis summarized in Table 4. Mixed-design repeated-measures ANOVA demonstrated significant main effects of Time (*F* = 151.757, *P* < 0.001) and Group (*F* = 5.220, *P* = 0.007) for TPI, whereas the Group-by-Time interaction did not reach statistical significance (*F* = 2.013, *P* = 0.069). Thus, TPI showed significant perioperative variation and an overall group effect, whereas the temporal trajectories did not differ significantly among the three groups. Although baseline TPI was comparable among the three groups (*P* = 0.962), TPI values in the Hypertension group were descriptively higher from T1 through T5, which may have contributed to the significant overall Group effect. For SBP, a significant Time effect was observed (*F* = 72.121, *P* < 0.001), whereas neither the Group effect (*P* = 0.291) nor the Group-by-Time interaction (*P* = 0.619) was significant. DBP showed a significant Time effect (*F* = 116.854, *P* < 0.001) and a significant Group-by-Time interaction (F = 2.181, *P* = 0.030), with no significant overall Group effect (*P* = 0.627). HR showed significant effects of Time (*F* = 99.918, *P* < 0.001), Group (*F* = 5.453, *P* = 0.006), and the Group-by-Time interaction (*F* = 3.307, *P* = 0.002).

**Figure 2:**
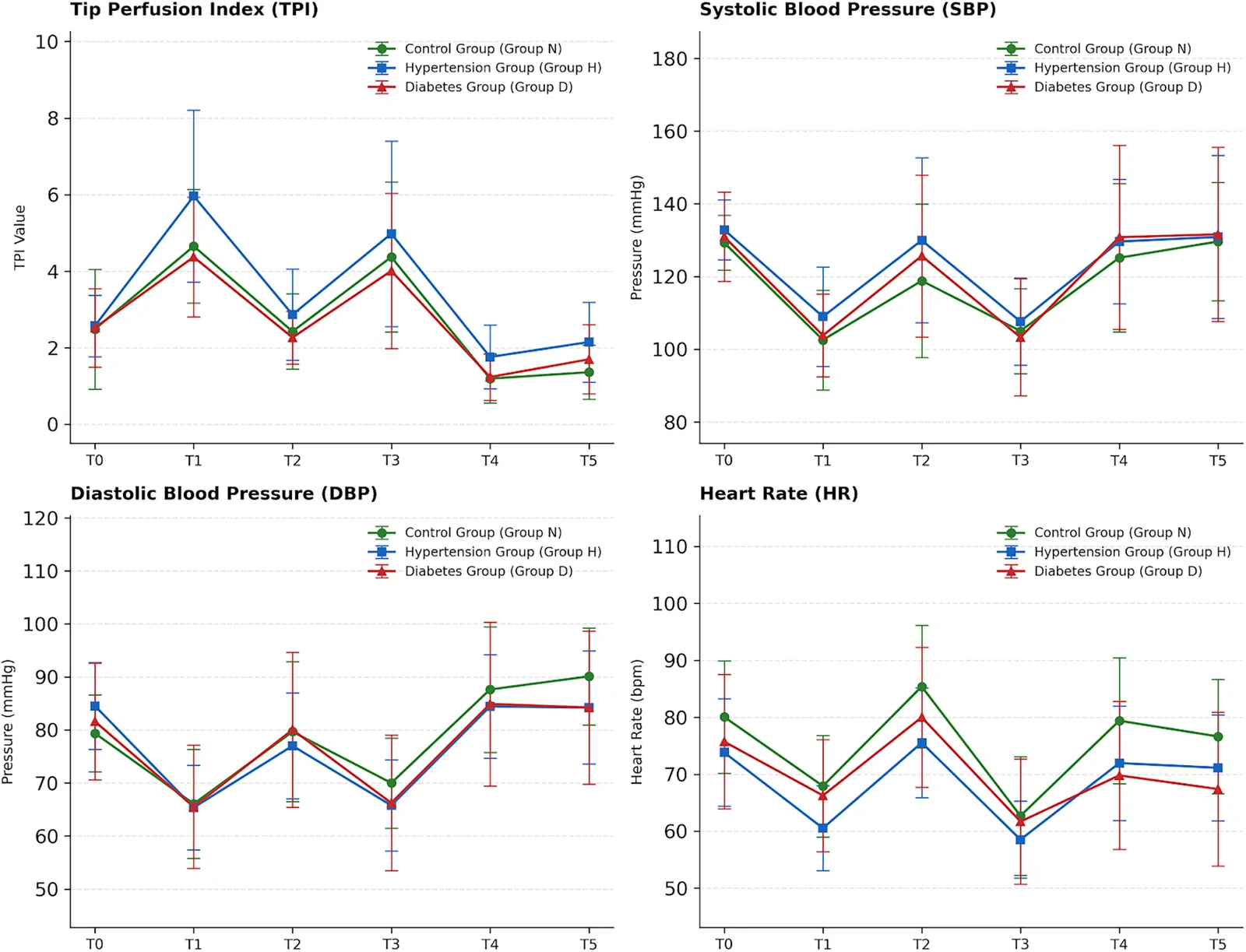
Dynamic trends of the TPI and systemic hemodynamic parameters across perioperative time points in the three study groups.

**Table 3:** Descriptive profiles of the tip perfusion index and hemodynamic parameters across perioperative time points.

Parameter/time point	Control Group (*n* = 30)	Hypertension Group (*n* = 30)	Diabetes Group (*n* = 30)	Statistical test values	*P* value
TPI $\left(\bar{x}\pm s\right)$					
T0 (Baseline)	2.49 ± 1.57	2.57 ± 0.80	2.52 ± 1.02	0.039	0.962
T1 (Pre-intubation)	4.65 ± 1.48a	5.96 ± 2.25a	4.37 ± 1.56a	6.719	0.002
T2 (Post-intubation)	2.42 ± 0.98b	2.86 ± 1.19b	2.26 ± 0.69b	3.040	0.053
T3 (Pre-pneumoperitoneum)	4.37 ± 1.96a	4.98 ± 2.43ab	4.01 ± 2.03a	1.569	0.214
T4 (Post-pneumoperitoneum 1 min)	1.19 ± 0.64abc	1.76 ± 0.83abc	1.23 ± 0.61abc	6.061	0.003
T5 (Post-pneumoperitoneum 5 min)	1.36 ± 0.71abc	2.15 ± 1.04bc	1.70 ± 0.90abc	5.838	0.004
SBP ($\bar{x}\pm s$, mmHg)					
T0 (Baseline)	129.30 ± 7.53	132.87 ± 8.23	130.97 ± 12.23	1.047	0.355
T1 (Pre-intubation)	102.57 ± 13.70a	108.97 ± 13.66a	103.80 ± 11.36a	2.062	0.133
T2 (Post-intubation)	118.83 ± 21.08ab	129.97 ± 22.68b	125.67 ± 22.28b	1.949	0.149
T3 (Pre-pneumoperitoneum)	105.03 ± 11.69a	107.63 ± 11.98a	103.30 ± 16.09a	0.794	0.455
T4 (Post-pneumoperitoneum 1 min)	125.17 ± 20.45bc	129.63 ± 17.15bc	130.83 ± 25.31bc	0.593	0.555
T5 (Post-pneumoperitoneum 5 min)	129.63 ± 16.28bc	130.90 ± 22.39bc	131.60 ± 23.92bc	0.067	0.935
DBP ($\bar{x}\pm s$, mmHg)					
T0 (Baseline)	79.37 ± 7.24	84.53 ± 8.20	81.60 ± 10.98	2.515	0.087
T1 (Pre-intubation)	66.07 ± 10.26a	65.37 ± 7.98a	65.50 ± 11.61a	0.041	0.960
T2 (Post-intubation)	79.67 ± 13.19b	77.00 ± 9.97ab	80.03 ± 14.60b	0.507	0.604
T3 (Pre-pneumoperitoneum)	70.00 ± 8.49ab	65.77 ± 8.60a	66.23 ± 12.78a	1.568	0.214
T4 (Post-pneumoperitoneum 1 min)	87.63 ± 11.85abc	84.43 ± 9.78bc	84.90 ± 15.46bc	0.566	0.570
T5 (Post-pneumoperitoneum 5 min)	90.10 ± 9.15abc	84.23 ± 10.66bc	84.23 ± 14.44bc	2.543	0.084
HR ($\bar{x}\pm s$, bpm)					
T0 (Baseline)	80.07 ± 9.88	73.83 ± 9.43	75.73 ± 11.78	2.824	0.065
T1 (Pre-intubation)	67.93 ± 8.92a	60.53 ± 7.44a	66.27 ± 9.83a	5.855	0.004
T2 (Post-intubation)	85.40 ± 10.78ab	75.53 ± 9.61b	80.00 ± 12.30b	6.108	0.003
T3 (Pre-pneumoperitoneum)	62.70 ± 10.41ab	58.53 ± 6.75a	61.70 ± 10.97ab	1.552	0.218
T4 (Post-pneumoperitoneum 1 min)	79.40 ± 11.03bc	71.97 ± 10.04bc	69.80 ± 12.98c	5.833	0.004
T5 (Post-pneumoperitoneum 5 min)	76.63 ± 10.03bc	71.13 ± 9.28bc	67.40 ± 13.51ac	5.259	0.007

a *P* < 0.05 versus T0; b *P* < 0.05 versus T1; c *P* < 0.05 versus T3, based on prespecified within-group comparisons with Bonferroni adjustment for multiple testing. T1 and T3 served as the immediate pre-stimulus reference values for endotracheal intubation and pneumoperitoneum, respectively.

**Table 4:** Repeated-measures analysis of TPI and hemodynamic parameters across perioperative time points.

Parameter	Effect	*F*	*P* value
TPI	Group	5.22	0.007
TPI	Time	151.757	<0.001
TPI	Group × Time	2.013	0.069
SBP (mmHg)	Group	1.251	0.291
SBP (mmHg)	Time	72.121	<0.001
SBP (mmHg)	Group × Time	0.778	0.619
DBP (mmHg)	Group	0.469	0.627
DBP (mmHg)	Time	116.854	<0.001
DBP (mmHg)	Group × Time	2.181	0.030
HR (bpm)	Group	5.453	0.006
HR (bpm)	Time	99.918	<0.001
HR (bpm)	Group × Time	3.307	0.002

Group was treated as the between-subject factor and Time as the within-subject factor. Greenhouse-Geisser correction was applied to the Time and Group × Time effects where appropriate.

In the planned within-group comparisons with Bonferroni adjustment, TPI decreased significantly at T2 relative to T1 following endotracheal intubation and at T4 and T5 relative to T3 following pneumoperitoneum in all three groups. Conversely, SBP, DBP, and HR increased significantly at T2 relative to T1 and at T4 and T5 relative to T3 in all three groups. The higher TPI values observed at T1 and T3 relative to T0 occurred before the corresponding noxious stimuli and were therefore not interpreted as stimulus-evoked stress responses. Because the predefined response thresholds were parameter specific and no independent reference standard for surgical stress was available, these temporal findings were not interpreted as evidence that TPI was more sensitive than conventional hemodynamic monitoring.

### 3.4 Stress hormone profiles and correlation analysis

The plasma concentrations of stress biomarkers—specifically E, NE, and COR—measured from T0 to T4 are illustrated in Figure 3. These neuroendocrine markers showed dynamic perioperative changes across the measured time points. Given the exploratory nature and small size of the hormone subcohort, these profiles were interpreted descriptively rather than as definitive evidence of between-group differences.

**Figure 3:**
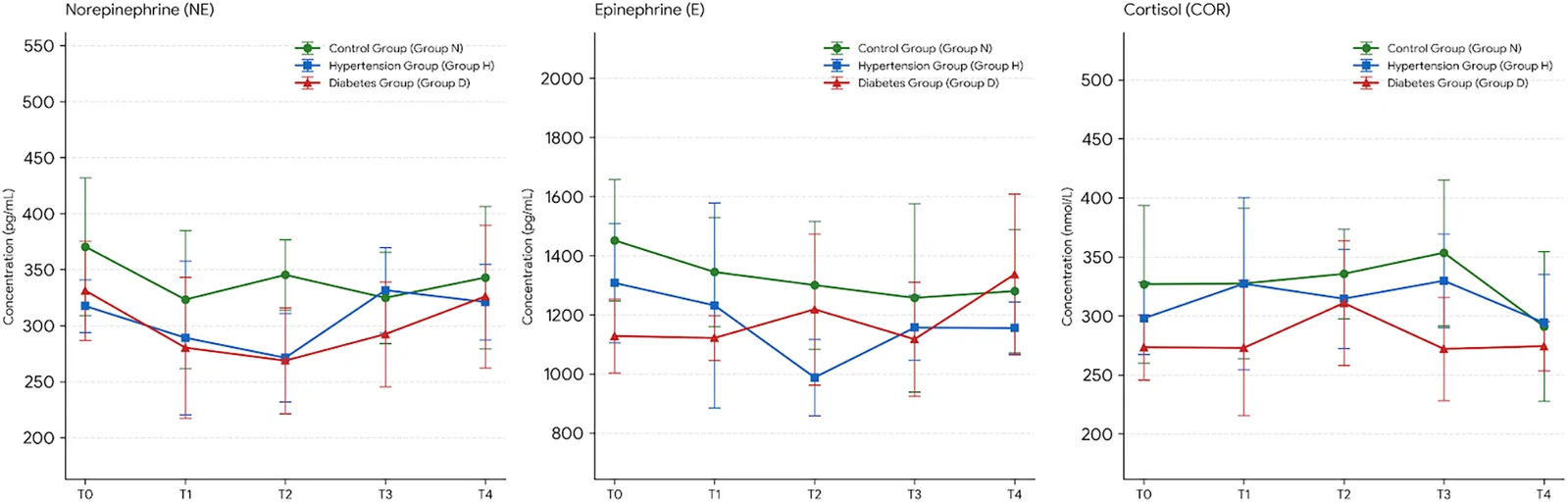
Dynamic trends of plasma stress hormone concentrations across perioperative time points in the three study groups.

Table 5 presents the repeated-measures correlation analyses accounting for within-participant dependence. TPI was significantly and inversely associated with SBP, DBP, and HR in all three groups (all *P* < 0.001). Significant inverse associations between TPI and E and NE were also observed across all groups (all *P* < 0.05). For COR, significant inverse associations were observed in the Control and Hypertension groups (both *P* < 0.001), whereas the association in the Diabetes group did not reach statistical significance (r-rm = −0.422, *P* = 0.057).

**Table 5:** Repeated-measures correlation analysis between tip perfusion index and hemodynamic parameters or stress hormones.

	Control Group		Hypertension Group		Diabetes Group	
	r_rm	*P*	r_rm	*P*	r_rm	*P*
Hemodynamic Parameters (30 patients per group)						
SBP	−0.598	<0.001	−0.346	<0.001	−0.508	<0.001
DBP	−0.703	<0.001	−0.513	<0.001	−0.605	<0.001
HR	−0.490	<0.001	−0.494	<0.001	−0.308	<0.001
Stress Hormones (5 patients per group)						
E	−0.752	<0.001	−0.728	<0.001	−0.437	0.048
NE	−0.747	<0.001	−0.515	0.017	−0.468	0.032
COR	−0.735	<0.001	−0.756	<0.001	−0.422	0.057

Hemodynamic analyses included 30 patients per group. Stress hormone analyses included a randomly selected subcohort of 5 patients per group, with measurements obtained at five perioperative time points from T0 through T4.

### 3.5 Positive stress response rates and response times

The incidence of positive stress responses and the time elapsed until criteria were met are summarized in Table 6 and visualized in Figure 4. During tracheal intubation, all groups reached the positive TPI criteria (100%), with no statistically significant differences observed among the three groups (*P* = 1.000). The response rates and the time taken to reach positive stress criteria for traditional hemodynamic parameters including SBP, DBP, and HR also showed no statistically significant differences among the groups (*P* > 0.05).

**Table 6:** Comparison of stress-positive rates and response times to stimuli among the three groups.

Outcome/parameter	Control Group	Hypertension Group	Diabetes Group	Statistical test values	*P* value
Tracheal Intubation					
Positive Stress Response Rate [n(%)]					
TPI	30 (100.00%)	30 (100.00%)	30 (100.00%)	—	1.000
SBP	22 (73.33%)	14 (46.67%)	14 (46.67%)	5.760	0.056
DBP	20 (66.67%)	12 (40.00%)	15 (50.00%)	4.364	0.113
HR	25 (83.33%)	17 (56.67%)	19 (63.33%)	5.291	0.071
Response Time to Criteria ($\bar{x}\pm s$, seconds)					
TPI	8.20 ± 4.85 (*n* = 30)	8.20 ± 3.79 (*n* = 30)	9.40 ± 4.70 (*n* = 30)	0.720	0.489
SBP	35.95 ± 8.17 (*n* = 22)	42.57 ± 14.29 (*n* = 14)	31.14 ± 16.31 (*n* = 14)	2.890	0.066
DBP	33.90 ± 7.21 (*n* = 20)	39.00 ± 12.76 (*n* = 12)	31.07 ± 15.72 (*n* = 15)	1.499	0.235
HR	24.48 ± 13.85 (*n* = 25)	21.82 ± 10.35 (*n* = 17)	20.79 ± 13.22 (*n* = 19)	0.493	0.613
Pneumoperitoneum					
Positive Stress Response Rate [n(%)]					
TPI	30 (100.00%)	30 (100.00%)	30 (100.00%)	—	1.000
SBP	20 (66.67%)	23 (76.67%)	21 (70.00%)	0.757	0.685
DBP	21 (70.00%)	23 (76.67%)	23 (76.67%)	0.467	0.792
HR	13 (43.33%)	14 (46.67%)	11 (36.67%)	0.638	0.727
Response Time to Criteria ($\bar{x}\pm s$, seconds)					
TPI	6.50 ± 3.71 (*n* = 30)	7.83 ± 4.08 (*n* = 30)	11.10 ± 5.23 (*n* = 30)	8.721	< 0.001
SBP	47.70 ± 13.11 (*n* = 20)	48.74 ± 14.08 (*n* = 23)	60.90 ± 37.63 (*n* = 21)	1.925	0.155
DBP	48.62 ± 18.15 (*n* = 21)	52.87 ± 25.05 (*n* = 23)	57.70 ± 31.42 (*n* = 23)	0.690	0.505
HR	41.62 ± 18.21 (*n* = 13)	53.00 ± 40.11 (*n* = 14)	43.55 ± 27.56 (*n* = 11)	0.537	0.589

Data are presented as *n* (%) or mean ± standard deviation, as appropriate. Each study group included 30 participants. Positive-response rates were calculated using all 30 participants in each group. Response times were calculated only among participants who reached the predefined response threshold; the responder sample size for each response-time estimate is shown in parentheses within the corresponding table cell. TPI and HR were monitored continuously, whereas SBP and DBP were measured intermittently; therefore, cross-parameter comparisons of response time should be interpreted cautiously.

**Figure 4:**
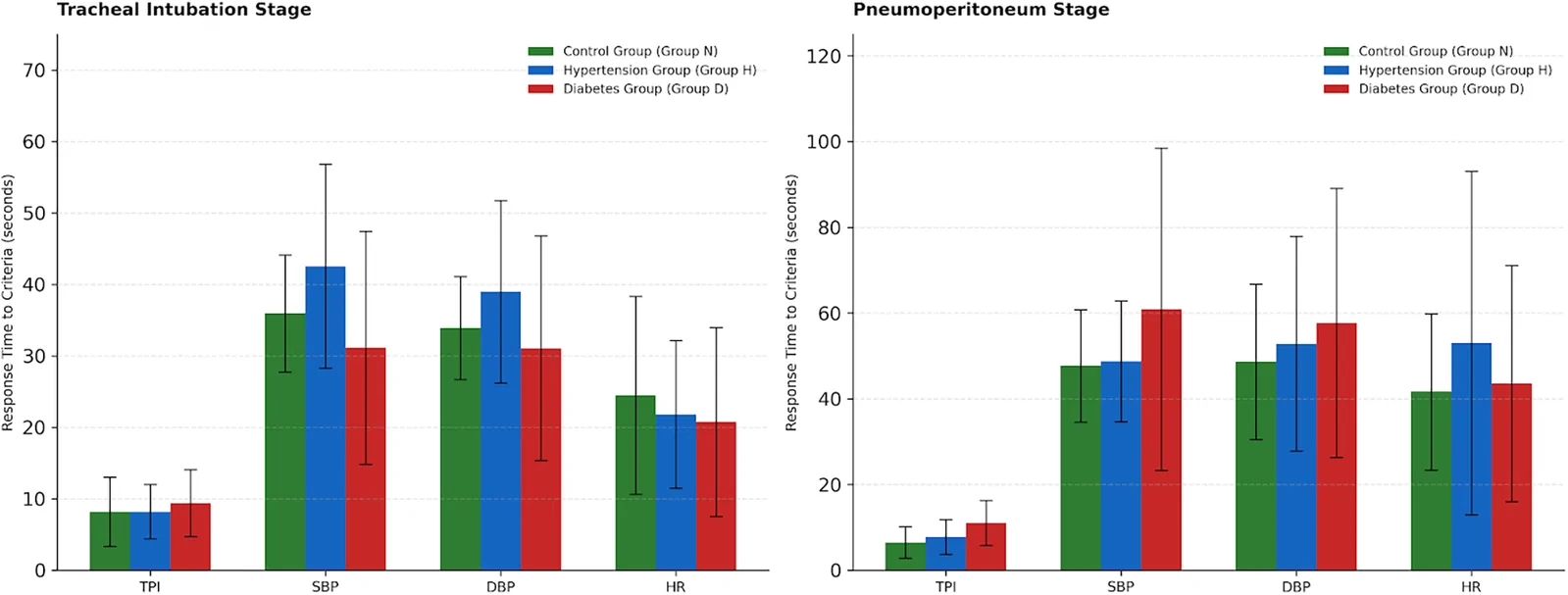
Positive stress response rates and response times across perioperative time points in the three study groups.

Positive-response status was determined relative to the corresponding immediate pre-stimulus reference value, using T1 for endotracheal intubation and T3 for pneumoperitoneum. Response time was defined as the interval from the onset of the corresponding stimulus to the first attainment of the predefined response threshold. Because TPI and HR were monitored continuously whereas SBP and DBP were obtained intermittently, cross-parameter comparisons of response time were interpreted cautiously and were considered descriptive rather than direct measures of relative temporal sensitivity. Response-time summaries were calculated only among participants who reached the predefined threshold for the corresponding parameter and stimulus; therefore, the denominators for these analyses varied according to the responder counts shown in Table 6. Participants who did not reach the threshold were not included in the response-time calculations. During the pneumoperitoneum stage, all participants met the predefined TPI-positive response criterion (100%), resulting in no between-group variability in this binary outcome. Because the predefined response thresholds were parameter specific, the positive-response rates were considered descriptive outcomes and were not used as a direct measure of comparative sensitivity across TPI, SBP, DBP, and HR. The response time to reach the TPI criterion differed significantly among the three groups (*P* < 0.001), with the longest mean response time observed in the Diabetes group. No significant between-group differences in response times were observed for SBP, DBP, or HR (all *P* > 0.05).

## 4 Discussion

### 4.1 Summary of main findings

This study characterized perioperative TPI responses in patients with hypertension and type 2 diabetes undergoing laparoscopic surgery. TPI showed clear peri-stimulus changes during endotracheal intubation and pneumoperitoneum, and all participants met the predefined TPI-positive response criterion during both stimuli. However, this 100% response rate represents a ceiling effect and should not be interpreted as evidence of superior sensitivity over conventional hemodynamic monitoring. The inverse associations between TPI and hemodynamic parameters and major stress hormones further support a physiological relationship between peripheral perfusion and perioperative stress responses.

Notably, the longitudinal and threshold-based analyses captured different aspects of the response. Group × Time interactions were significant for DBP and HR, but not for TPI or SBP, whereas TPI response time during pneumoperitoneum differed significantly among groups. Therefore, the longer TPI response time observed in the Diabetes group should be interpreted as a difference in threshold-crossing latency rather than as evidence of a distinct overall longitudinal TPI trajectory. The significant overall Group effect for TPI should therefore be interpreted as an average between-group difference across repeated measurements rather than as evidence of different temporal trajectories, particularly because the Group × Time interaction was not statistically significant.

### 4.2 Physiological basis of TPI responses to surgical stress

The observed peri-stimulus changes in TPI are physiologically consistent with the responsiveness of the peripheral microvasculature to sympathetic nervous system (SNS) activation (23). Unlike macro-hemodynamic parameters such as systemic blood pressure and heart rate, which are influenced by multiple compensatory mechanisms and baroreceptor regulation, peripheral vascular tone is closely modulated by sympathetic activity (24). During noxious stimulation such as tracheal intubation or pneumoperitoneum, sympathetic activation and catecholamine release can induce peripheral vasoconstriction, thereby reducing peripheral perfusion and TPI values (25, 26).

These physiological characteristics provide a plausible basis for the marked TPI changes observed around surgical stimulation in the present study (27, 28). However, because the predefined TPI-positive criterion was reached by all participants, the present data do not establish superior diagnostic sensitivity relative to conventional hemodynamic parameters. Rather, TPI may provide complementary information regarding peripheral vascular responses during perioperative stress (29), while its comparative monitoring performance requires further evaluation using validated reference standards.

The higher TPI values observed at T1 and T3 relative to T0 should be interpreted in the context of anesthesia rather than as stress-related responses. T0 was recorded before anesthesia induction, whereas T1 and T3 were obtained after induction and during maintenance of general anesthesia. Reduced sympathetic vascular tone and anesthesia-related peripheral vasodilation may have contributed to the higher pre-stimulus TPI values. This interpretation further supports the use of T1 and T3, rather than T0, as the immediate reference values for evaluating responses to endotracheal intubation and pneumoperitoneum, respectively.

### 4.3 Influence of diabetes on peripheral vascular response dynamics

The longer TPI response time observed in the Diabetes group during pneumoperitoneum may reflect altered peripheral vascular response dynamics associated with type 2 diabetes. Because propofol and remifentanil can influence sympathetic and hemodynamic responses, potential between-group differences in anesthetic drug exposure were also considered. No statistically significant between-group differences in the recorded doses of either agent were detected at the assessed perioperative time points. Although this finding reduces concern regarding a major between-group imbalance in anesthetic drug exposure, residual confounding related to individual anesthetic requirements or anesthetic depth cannot be completely excluded. Endothelial dysfunction, reduced nitric oxide bioavailability, and autonomic abnormalities are plausible mechanisms described in diabetes; however, these were not directly assessed in the present study (30–33). Therefore, the observed delay should be interpreted as a clinical difference in response timing rather than as direct evidence of diabetic microangiopathy or autonomic neuropathy.

### 4.4 Clinical implications

The integration of TPI monitoring into perioperative care may provide complementary information on peripheral vascular responses, particularly in patients with comorbidities. In the present study, TPI showed clear peri-stimulus changes during endotracheal intubation and pneumoperitoneum; however, the 100% positive-response rate precludes inference of superior sensitivity relative to conventional hemodynamic parameters. Accordingly, TPI should currently be regarded as an adjunct to conventional monitoring rather than as an independently validated early-warning tool. Further studies using validated reference standards are needed to determine whether TPI-guided interpretation can improve the timing or precision of anesthetic and analgesic management (34, 35).

The observed difference in TPI response time in patients with diabetes may have potential implications for individualized perioperative monitoring. Because the underlying mechanisms were not directly assessed, the prolonged response time should be interpreted cautiously as a difference in response dynamics rather than as evidence of a specific microvascular defect. Clinicians may therefore consider diabetes-related comorbidity when interpreting peripheral perfusion changes during surgery (36). At present, TPI may be regarded as a complementary monitoring variable, while its role in guiding individualized anesthetic or analgesic management requires further prospective validation.

### 4.5 Limitations

Several limitations should be acknowledged. First, this was a single-center study with a modest sample size, and the neuroendocrine analysis was based on an exploratory subcohort of only five patients per group. Second, residual confounding from medication use, diabetes duration, antidiabetic treatment, and anesthetic depth cannot be completely excluded. Although no statistically significant between-group differences were detected in the recorded propofol and remifentanil doses at the assessed perioperative time points, individual variability in anesthetic requirements and actual anesthetic depth may still have influenced sympathetic and hemodynamic responses. Although no participant was excluded on the basis of the preliminary deep-breath maneuver, this prespecified screening criterion could theoretically limit applicability to patients with absent peripheral perfusion responsiveness. Third, the positive-response thresholds were parameter specific, and response-time analyses included only participants who reached the predefined threshold, which may introduce selection bias. Fourth, TPI and HR were monitored continuously, whereas SBP and DBP were measured intermittently, limiting direct cross-parameter comparisons of response time. In addition, catecholamine concentrations were measured using ELISA rather than chromatographic methods such as HPLC or LC-MS/MS. Although ELISA was suitable for the exploratory within-study analyses, its analytical specificity may be lower than that of chromatographic methods; therefore, the absolute catecholamine concentrations and their associations with TPI should be interpreted cautiously. Detailed ELISA kit identifiers and lot-specific precision data were also unavailable from the archived records. Finally, the study was limited to laparoscopic surgery, and TPI remains an indirect surrogate of sympathetic activation. Larger multicenter studies are needed to validate these findings and further clarify the clinical role of TPI.

## 5 Conclusions

This study demonstrates that TPI exhibits consistent peri-stimulus changes during laparoscopic surgery and may provide complementary information on peripheral vascular responses to surgical stress. The longer TPI response time observed in patients with diabetes suggests potential differences in peripheral vascular response dynamics associated with metabolic comorbidity. Further studies using validated reference standards and larger, diverse populations are needed to determine the comparative monitoring value and clinical utility of TPI in perioperative care.

## Data Availability

The datasets utilized and/or analyzed during the current study are available from the corresponding author upon reasonable request. Requests to access the datasets should be directed to 26901224@hebmu.edu.cn.

## References

[1] Del Rio JM Nicoara A Swaminathan M. Neuroendocrine stress response: implications for cardiac surgery-associated acute kidney injury. Rom J Anaesth Intensive Care. (2017) 24(1):57–63. 10.21454/rjaic.7518.241.hav 28913500 PMC5555430

[2] Silva GN Brandão VGA Perez MV Blum K Lewandrowski KU Fiorelli RKA. Neuroinflammatory approach to surgical trauma: biomarkers and mechanisms of immune and neuroendocrine responses. J Pers Med. (2024) 14(8):829. 10.3390/jpm14080829 39202020 PMC11355628

[3] Roxburgh BH Cotter JD Campbell HA Reymann U Wilson LC Gwynne-Jones D. Physiological relationship between cardiorespiratory fitness and fitness for surgery: a narrative review. Br J Anaesth. (2023) 130(2):122–32. 10.1016/j.bja.2022.10.039 36529576

[4] Johansson JK Puukka PJ Virtanen R Jula AM. Beat-to-beat, ambulatory hour-to-hour, and home day-to-day variabilities in blood pressure, pulse pressure, and heart rate in comparison with each other and with target-organ damage. Blood Press Monit. (2015) 20(3):113–20. 10.1097/MBP.0000000000000101 25943466

[5] De Louche CD Mandal M Fernandes L Lawson J Bicknell CD Pouncey AL. Heart rate variability as a dynamic marker of surgeons’ stress during vascular surgery. BJS Open. (2024) 8(5):zrae097. 10.1093/bjsopen/zrae097 39270744 PMC11398904

[6] Demir ZA Bingül ES Dost B Talih G Aykut A Aydın ME. Foundations and advancements in hemodynamic monitoring: part I-elements of hemodynamics. Turk J Anaesthesiol Reanim. (2025) 53(3):87–97. 10.4274/TJAR.2025.251925 40150830 PMC12123657

[7] Woods J Turner J Hughes A Davies G Grier G. The non-haemorrhagic vagal response to trauma: a review of hypotensive and bradycardic responses to injury in the absence of bleeding. Eur J Trauma Emerg Surg. (2024) 50(5):1995–2004. 10.1007/s00068-024-02648-y 39230585 PMC11599317

[8] Orellana Plaza I Dankelman J Bakker J. Peripheral perfusion noninvasive monitoring technologies—a literature and patent review. J Clin Monit Comput. (2026) 40(3):673–86. 10.1007/s10877-026-01412-4 41627642 PMC13194216

[9] Aissaoui Y Bouchama A Abouelkemhe C Enourhbi A Bencharfa B Didi M. The reliability of the plethysmographic perfusion index for detecting fluid responsiveness in critically ill patients. J Intensive Med. (2025) 6(1):34–41. 10.1016/j.jointm.2025.07.004 41736778 PMC12925886

[10] Kasai K Kawasaki T. Peripheral perfusion index as a marker of hypoperfusion in heart failure. Cardiology. (2026:1–6). 10.1159/000550873 41662304

[11] Højlund J Petersen DR Agerskov M Foss NB. The peripheral perfusion index discriminates haemodynamic responses to induction of general anaesthesia. J Clin Monit Comput. (2023) 37(6):1533–40. 10.1007/s10877-023-01035-z PMC10651705 37289351

[12] Høiseth LØ Hisdal J Hoff IE Hagen OA Landsverk SA Kirkebøen KA. Tissue oxygen saturation and finger perfusion index in central hypovolemia: influence of pain. Crit Care Med. (2015) 43(4):747–56. 10.1097/CCM.0000000000000766 25513787

[13] Cao S Kan M Jia Y Wang C Wang T. Index of consciousness monitoring may effectively predict and prevent circulatory stress induced by endotracheal intubation under general anesthesia: a prospective randomized controlled trial. BMC Anesthesiol. (2024) 24(1):316. 10.1186/s12871-024-02701-8 39243003 PMC11378600

[14] Uemura N Nomura M Inoue S Endo J Kishi S Saito K. Changes in hemodynamics and autonomic nervous activity in patients undergoing laparoscopic cholecystectomy: differences between the pneumoperitoneum and abdominal wall-lifting method. Endoscopy. (2002) 34(8):643–50. 10.1055/s-2002-33252 12173086

[15] Skorus U Rapacz K Kenig J. The significance of comorbidity burden among older patients undergoing abdominal emergency or elective surgery. Acta Chir Belg. (2021) 121(6):405–12. 10.1080/00015458.2020.1816671 32873179

[16] Durante A Mazzapicchi A Baiardo Redaelli M. Systemic and cardiac microvascular dysfunction in hypertension. Int J Mol Sci. (2024) 25(24):13294. 10.3390/ijms252413294 39769057 PMC11677602

[17] Maruhashi T Higashi Y. Pathophysiological association between diabetes mellitus and endothelial dysfunction. Antioxidants (Basel). (2021) 10(8):1306. 10.3390/antiox10081306 34439553 PMC8389282

[18] Guo X Xing Y Jin W. Role of ADMA in the pathogenesis of microvascular complications in type 2 diabetes mellitus. Front Endocrinol (Lausanne). (2023) 14:1183586. 10.3389/fendo.2023.1183586 37152974 PMC10160678

[19] Li SQ Luo BR Wang BG. Article in Chinese. Zhonghua Yi Xue Za Zhi. (2008) 88(17):1177–80. 18844111

[20] Takahashi S Tanaka M Toyooka H. Fentanyl pretreatment does not impair the reliability of an epinephrine-containing test dose during propofol-nitrous oxide anesthesia. Anesth Analg. (1999) 89(3):743–7. 10.1097/00000539-199909000-00040 10475317

[21] Mowafi HA Ismail SA Shafi MA Al-Ghamdi AA. The efficacy of perfusion index as an indicator for intravascular injection of epinephrine-containing epidural test dose in propofol-anesthetized adults. Anesth Analg. (2009) 108(2):549–53. 10.1213/ane.0b013e31818fc35b 19151286

[22] Atef HM Fattah SA Gaffer ME Al Rahman AA. Perfusion index versus non-invasive hemodynamic parameters during insertion of i-gel, classic laryngeal mask airway and endotracheal tube. Indian J Anaesth. (2013) 57(2):156–62. 10.4103/0019-5049.111843 23825815 PMC3696263

[23] Højlund J Agerskov M Clemmesen CG Hvolris LE Foss NB. The peripheral perfusion index tracks systemic haemodynamics during general anaesthesia. J Clin Monit Comput. (2020) 34(6):1177–84. 10.1007/s10877-019-00420-x 31705432

[24] Wajima Z Shiga T Imanaga K. Does pneumoperitoneum affect perfusion index and pleth variability index in patients receiving combined epidural and general anesthesia? Biosci Trends. (2017) 11(6):667–74. 10.5582/bst.2017.01253 29249773

[25] Jansen AS Nguyen XV Karpitskiy V Mettenleiter TC Loewy AD. Central command neurons of the sympathetic nervous system: basis of the fight-or-flight response. Science. (1995) 270(5236):644–6. 10.1126/science.270.5236.644 7570024

[26] Ivascu R Torsin LI Hostiuc L Nitipir C Corneci D Dutu M. The surgical stress response and anesthesia: a narrative review. J Clin Med. (2024) 13(10):3017. 10.3390/jcm13103017 38792558 PMC11121777

[27] Tabi M Padkins M Burstein B Younis A Asher E Bennett C. Association of shock index with echocardiographic parameters in cardiac intensive care unit. J Crit Care. (2024) 79:154445. 10.1016/j.jcrc.2023.154445 37890356

[28] van Genderen ME Bartels SA Lima A Bezemer R Ince C Bakker J. Peripheral perfusion index as an early predictor for central hypovolemia in awake healthy volunteers. Anesth Analg. (2013) 116(2):351–6. 10.1213/ANE.0b013e318274e151 23302972

[29] Aljuri N Cohen RJ. Theoretical considerations in the dynamic closed-loop baroreflex and autoregulatory control of total peripheral resistance. Am J Physiol Heart Circ Physiol. (2004) 287(5):H2252–73. 10.1152/ajpheart.00489.2003 15231503

[30] Syed NA Bhatti A John P. Molecular link between glo-1 expression and markers of hyperglycemia and oxidative stress in vascular complications of type 2 diabetes mellitus. Antioxidants (Basel). (2023) 12(9):1663. 10.3390/antiox12091663 37759966 PMC10525326

[31] Lu X Xie Q Pan X Zhang R Zhang X Peng G. Type 2 diabetes mellitus in adults: pathogenesis, prevention and therapy. Signal Transduct Target Ther. (2024) 9(1):262. 10.1038/s41392-024-01951-9 39353925 PMC11445387

[32] Kilo S Berghoff M Hilz M Freeman R. Neural and endothelial control of the microcirculation in diabetic peripheral neuropathy. Neurology. (2000) 54(6):1246–52. 10.1212/wnl.54.6.1246 10746593

[33] Meyer MF Lieps D Schatz H Pfohl M. Impaired flow-mediated vasodilation in type 2 diabetes: lack of relation to microvascular dysfunction. Microvasc Res. (2008) 76(1):61–5. 10.1016/j.mvr.2008.03.001 18448131

[34] Elshal MM Hasanin AM Mostafa M Gamal RM. Plethysmographic peripheral perfusion index: could it be a new vital sign? Front Med (Lausanne). (2021) 8:651909. 10.3389/fmed.2021.651909 34660615 PMC8517109

[35] Lorente JV Jimenez I Ripollés-Melchor J Becerra A Wesselink W Reguant F. Intraoperative haemodynamic optimisation using the hypotension prediction index and its impact on tissular perfusion: a protocol for a randomised controlled trial. BMJ Open. (2022) 12(6):e051728. 10.1136/bmjopen-2021-051728 35654467 PMC9163532

[36] Jan Y-K Kelhofer N Tu T Mansuri O Onyemere K Dave S. Diagnosis, pathophysiology and management of microvascular dysfunction in diabetes mellitus. Diagnostics (Basel). (2024) 14(24):2830. 10.3390/diagnostics14242830 39767191 PMC11674805

